# *Atractylodes macrocephala* Koidz Alleviates Symptoms in Zymosan-Induced Irritable Bowel Syndrome Mouse Model through TRPV1, NaV1.5, and NaV1.7 Channel Modulation

**DOI:** 10.3390/nu16111683

**Published:** 2024-05-29

**Authors:** Na-Ri Choi, Woo-Gyun Choi, Jong-Hwan Lee, Joon Park, Yun-Tai Kim, Raju Das, Joo-Han Woo, Byung-Joo Kim

**Affiliations:** 1 Department of Longevity and Biofunctional Medicine, School of Korean Medicine, Pusan National University, Yangsan 50612, Republic of Korea; nariring@gmail.com (N.-R.C.); ak0510@hanmail.net (W.-G.C.); 2 Department of Korean Medical Science, School of Korean Medicine, Pusan National University, Yangsan 50612, Republic of Korea; 3 Department of Biomedical Engineering, College of Engineering, Dong-Eui University, Busan 47340, Republic of Korea; jonghwanlee@deu.ac.kr; 4 Division of Food Functionality, Korea Food Research Institute, Wanju-gun 55365, Republic of Korea; biosciencepark@gmail.com (J.P.); ytkim@kfri.re.kr (Y.-T.K.); 5 Department of Food Biotechnology, Korea University of Science & Technology, Daejeon 34113, Republic of Korea; 6 Department of Physiology, College of Medicine, Dongguk University, Gyeongju 38066, Republic of Korea; rajudasbgc@gmail.com

**Keywords:** gastrointestinal disease, traditional medicine, visceral hypersensitivity, quality of life, ion channel

## Abstract

(1) Background: Irritable bowel syndrome (IBS) is a common disease in the gastrointestinal (GI) tract. *Atractylodes macrocephala* Koidz (AMK) is known as one of the traditional medicines that shows a good efficacy in the GI tract. (2) Methods: We investigated the effect of AMK in a network pharmacology and zymosan-induced IBS animal model. In addition, we performed electrophysiological experiments to confirm the regulatory mechanisms related to IBS. (3) Results: Various characteristics of AMK were investigated using TCMSP data and various analysis systems. AMK restored the macroscopic changes and weight to normal. Colonic mucosa and inflammatory factors were reduced. These effects were similar to those of amitriptyline and sulfasalazine. In addition, transient receptor potential (TRP) V1, voltage-gated Na^+^ (NaV) 1.5, and NaV1.7 channels were inhibited. (4) Conclusion: These results suggest that AMK may be a promising therapeutic candidate for IBS management through the regulation of ion channels.

## 1. Introduction

*Atractylodes macrocephala* Koidz (AMK) plays a significant role as an essential traditional herbal remedy in China, Japan, Korea, and Thailand [1]. In accordance with the principles of Traditional Chinese Medicine, AMK possesses properties that promote spleen strengthening and dampness removal. This traditional perspective underscores its value in holistic healing systems [1]. AMK has a variety of pharmacological abilities such as anti-inflammatory activity, anti-oxidative activity, antiosteoporotic activity, antibacterial activity, anti-tumor activity, anti-obesity activity, and energy-enhancing metabolism [1]. In addition, in particular, it is used in the treatment of chronic diseases. Among them, it has been known to show the most prominent effect on gastrointestinal (GI) functions [1]. AMK regulates the movement of epithelial cells in the gut under the influence of polyamine and Rho mRNA, thereby promoting the recovery of intestinal wounds [2,3]. The polysaccharides component of AMK promotes rat activity and activates the ability of the gut microbiota [4]. It has also been known to help GI function by promoting the growth of gut microvilli related to the treatment of intestinal damage [5]. Other components of AMK, atractylenolide I and III and 4,15-epoxy-8βhydroxyasterolide, have been reported to have a protective effect on the GI tract by increasing muscle contraction [6].

Irritable bowel syndrome (IBS) is a common condition that occurs in the GI tract, and despite the high prevalence of IBS, its exact cause is still not well understood and current treatment approaches often focus on alleviating symptoms [7]. However, emerging research has shed light on the potential of traditional herbal medicines, such as AMK, to address the multifaceted nature of IBS [8]. IBS is a complex condition with various underlying factors, such as stress, gut dysbiosis, visceral hypersensitivity, and altered gut motility, which contribute to the uncomfortable and sometimes debilitating symptoms experienced by individuals with IBS [9]. Importantly, ion channels within the GI tract play a pivotal role in regulating various digestive processes, including secretion, absorption, motility, and visceral hypersensitivity. The dysregulation of these ion channels can contribute to IBS symptom development and persistence [2]. Recent research suggests that traditional medicines may be promising for alleviating IBS symptoms through ion channel modulation [10]. Therefore, this possibility has been investigated using a zymosan-induced IBS animal model that closely mimics the symptoms experienced by patients with IBS [11,12].

In this study, we assessed the effectiveness of AMK in this zymosan-induced IBS model and explored the mechanisms of its therapeutic effects. It is known that the animal model of IBS made by administering zymosan to the colon is similar to IBS, whose main symptom is diarrhea in humans [11,13]. By examining the effect of AMK on the zymosan-induced IBS model, this study aimed to confirm the potential of this traditional herbal medicine as a therapeutic agent for IBS. Additionally, we sought to unravel the underlying mechanisms, particularly ion channel modulation, which may offer valuable insights into the treatment of this complex GI disorder. This investigation could pave the way for novel approaches to IBS management and potentially improve the quality of life of patients affected by this condition.

## 2. Materials and Methods

### 2.1. Analysis of AMK Targets

Various information and proteins about the targets of AMK were obtained using the traditional Chinese medical systems pharmacology (TCMSP) and Uniprot data (https://www.uniprot.org/uniprot, accessed on 21 May 2023) [14,15].

### 2.2. Preparation of AMK Extract and High-Performance Liquid Chromatography (HPLC) Analysis

The AMK extract, sourced from the Korea Plant Extract Bank (Ochang, Chungbuk, Republic of Korea) was prepared using ethanol, as described in the previous study [15]. Eudesma-4(15),7(11)-dien-8-one (EDO) and Atractylenolide III (ATO III) were detected by HPLC (JASCO, Easton, MD, USA) [15]. A chromatographic analysis was conducted using a HPLC system comprising a binary pump, an auto sampler, a column oven, and a UV detector. This analysis used 50–65% acetonitrile for the first 0–35 min, followed by 65–100% acetonitrile for the next 35–50 min. A re-equilibration period of 5 min was allowed between sample injections [15].

### 2.3. Animal Experiments

**Figure 1:**
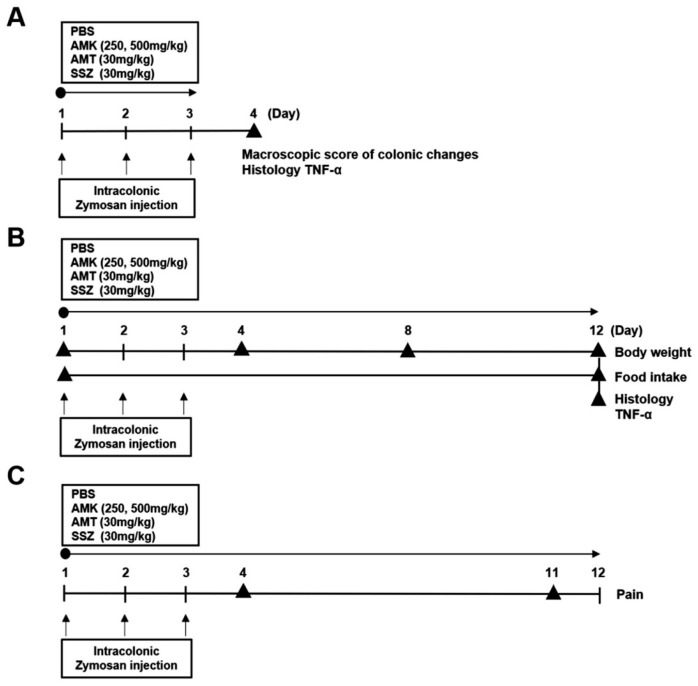
Animal experiment timeline. (**A**) Timeline for colonic changes. (**B**) Timeline for Body weight, Food intake and Histology. (**C**) Timeline for Pain behavior. According to the presented schedule, mice were subjected to various treatments including PBS, AMK extract (250 or 500 mg/kg), AMT (30 mg/kg), and SSZ (30 mg/kg).

Male C57/BL6 mice were used in this study (Samtako Bio, Osan, Republic of Korea). Colitis was induced by administering 0.1 mL 30 mg/mL zymosan (Sigma-Aldrich, St. Louis, MO, USA) into the colon using a flexible Zonde for three days. The mice were categorized into six distinct groups: (a) normal group (*n* = 19 mice), (b) PBS control group (*n* = 20), (c) amitriptyline (AMT) group (30 mg/kg, *n* = 18), (d) sulfasalazine (SSZ) group (30 mg/kg, *n* = 17), (e) AMK extract group (250 mg/kg, *n* = 21), and (f) AMK extract group (500 mg/kg, *n* = 20). The animal experimental methods are summarized in Figure 1.

### 2.4. Measurement of Body Weight Change and Food Intake

The body weights were checked on days 1, 4, 8, and 12 to monitor the changes in body weight. Additionally, the cumulative amount of food consumed over this period was calculated and recorded to assess food intake.

### 2.5. Observation of Changes in Colon and Stool Conditions

To assess zymosan-induced changes in the colon, we measured the colon weight and length, which were quantified as the distance between the cecal end and the anus. Additionally, stool conditions were evaluated by three researchers in a blinded manner. A scoring system based on the Bristol Stool scale was used to classify stool conditions into four levels: 0 (normal), 1 (moist), 2 (sticky), and 3 (diarrhea).

### 2.6. Histological Examination of the Colon

For the histological examination, biopsies were collected from the large intestine of the mice in each experimental group. The tissue sections were fixed, paraffin-embedded, stained using hematoxylin and eosin (H&E) for general tissue examination, and observed under a visible-light microscope (Nikon, Tokyo, Japan).

### 2.7. Tumor Necrosis Factor (TNF)-α Gene Expression Evaluation by Quantitative RT-qPCR

To evaluate the colitis-induced inflammation-related TNF-α gene expression, the total mRNA was extracted from the colonic tissue using TRIzol reagent (Invitrogen, Waltham, MA, USA). Next, cDNA was synthesized from the total RNA samples using a cDNA reverse transcription kit (M-MLV Reverse Transcriptase, Promega, Madison, WI, USA).

### 2.8. Pain-Related Behavior Measurement

Pain-related behavioral measures were quantified based on the procedures outlined in a previous study [16]. This assessment included movements, such as licking the abdomen, stretching the entire body, placing the abdomen on the floor, and arching by contracting the abdomen. To ensure the accuracy of these observations, two researchers independently checked the pain-related behaviors (10 min).

### 2.9. Plasmid Transfection

HEK293T cells were seeded in 12-well plates, and the following day, plasmids (1.5–2 g/well) containing human transient receptor potential (TRP) V1, TRPV4, TRPA1, or voltage-gated Na^+^ (NaV) 1.5/1.7 genes were transfected into the HEK293T cells using the transfection reagent (Thermo Fisher Scientific, Waltham, MA, USA). For visual confirmation of successful transfection, co-transfection with pEGFP-N1 (50–100 ng/well) was performed.

### 2.10. Electrophysiological Experiments

The whole-cell patch-clamp technique was used. Data acquisition was carried out using an Axopatch 200B amplifier (Molecular Devices, San Jose, CA, USA). To measure the TRP currents, the holding voltage was set to −60 mV and the ramp pulses ranged from −100 mV to 100 mV. For the NaV channels, the currents were measured from −120 mV to 0 mV, with −120 mV as the holding voltage. Details regarding the compositions of the internal and external solutions are provided in the previous study [10].

### 2.11. Statistical Analysis

Data are represented as mean ± SE. To assess variance, one-way analysis of variance (ANOVA) was conducted, and Dunnett’s multiple comparison test was employed. The software used for statistical analyses was GraphPad Prism 8. Statistical significance was set at *p* < 0.05.

## 3. Results

### 3.1. Active Compounds of AMK

In total, 55 potential active compounds were identified in AMK using the TCMSP [15] and 18 active compounds satisfied the absorption, distribution, metabolism, and excretion (ADME) criteria (Table 1).

### 3.2. Identification of 35 Compounds Related to GI Diseases in AMK

**Figure 2:**
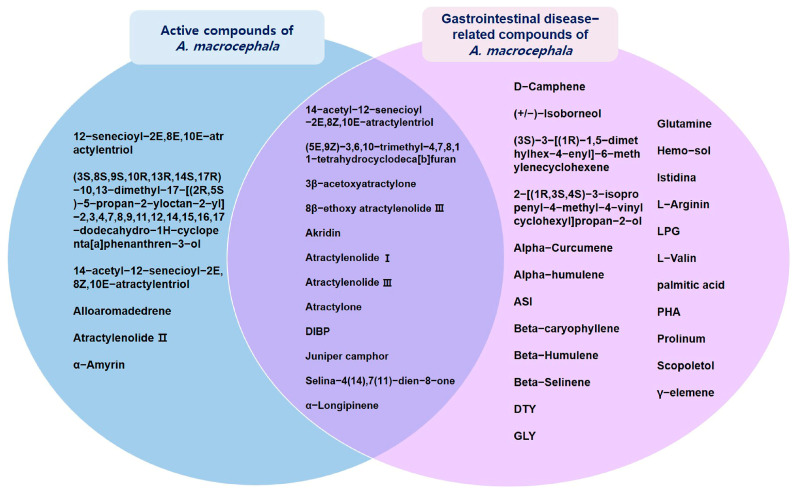
Interactions between active compounds from AMK and GI disease-related compounds.

The association between the AMK compound and target and disease was investigated. Overall, 35 compounds were involved in GI disease (Table 2), 12 of which were active compounds (Figure 2).

### 3.3. Eight Genes Are Included in Both IBS-Related Genes and AMK Target Genes

**Figure 3:**
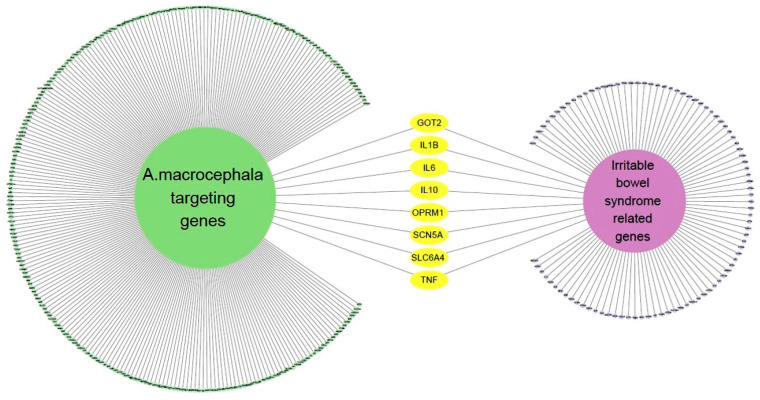
The network of IBS-related genes and AMK targeting genes.

We identified 100 genes associated with IBS by applying a confidence threshold of 0.40 for up to 100 proteins (Supplementary Material Table S1). Next, we made a network containing IBS-related genes and AMK target genes (Figure 3), which revealed the presence of eight genes common to both gene sets.

### 3.4. Relevance of AMK Compounds and IBS-Related Genes

**Figure 4:**
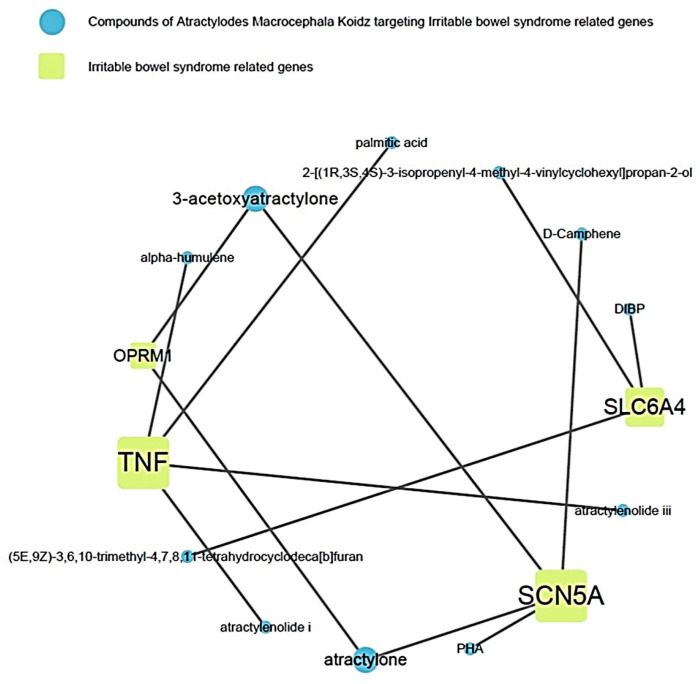
Relevance of AMK compounds and IBS-related genes.

This showed the relationship between the active compound of AMK and the target gene related to IBS (Figure 4). Among them, it showed that TNF and SCN5A were the most related to IBS.

### 3.5. Effects of AMK Extract on Zymosan-Induced Colonic Changes

**Figure 5:**
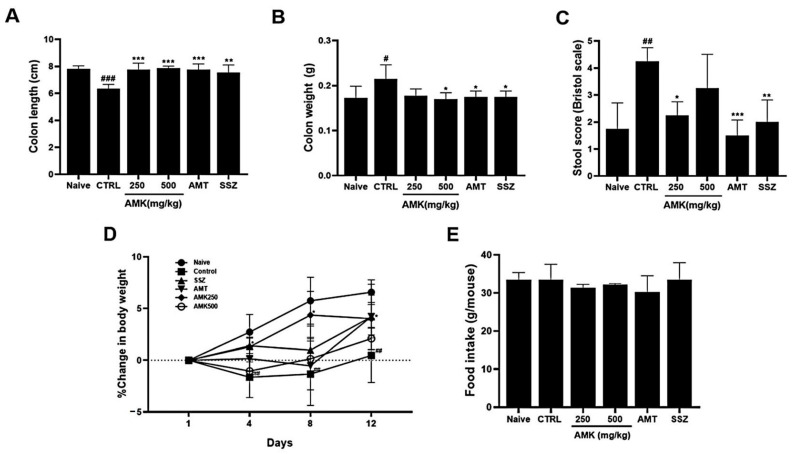
Effects of AMK extract on gut macroscopic changes, body weight, and food intake. Macroscopic evaluations were investigated in (**A**) colon length, (**B**) colon weight, and (**C**) stool score. Examined alterations in (**D**) body weight and assessed (**E**) food intake. The results are represented as mean ± SE. # *p* < 0.05, ## *p* < 0.01 and ### *p* < 0.001 denote statistical significance compared to naïve, while * *p* < 0.05, ** *p* < 0.01, and *** *p* < 0.001 indicate statistical significance relative to the control. CTRL: Control.

We examined the colon length and weight and stool conditions in the zymosan-induced mice following AMK extract administration. Notably, 30 mg/kg AMT (antidepressant drug) [17] and SSZ (anti-inflammatory drug) [18], both commonly used in clinical IBS treatment, served as positive controls. In colitis, the intestinal length is reduced and its weight increases [19]. The colon length of the zymosan-induced mice was smaller than that of naïve mice, indicating colitis induction. However, the colon length returned to normal levels in the oral AMK-extract-administered mice (7.82 ± 0.22, 6.34 ± 0.33, 7.77 ± 0.47, 7.87 ± 0.15, 7.76 ± 0.42, and 7.55 ± 0.55 cm in naïve, control (### *p* < 0.001), 250 mg/kg AMK extract (*** *p* < 0.001), 500 mg/kg AMK extract (*** *p* < 0.001), AMT (*** *p* < 0.001), and SSZ (** *p* < 0.01) mice, respectively; Figure 5A). The weight of the large intestine of the zymosan-induced mice also increased significantly, indicating colitis. However, the colon weight recovered in the oral AMK-extract-administered mice (0.173 ± 0.026, 0.215 ± 0.031, 0.178 ± 0.015, 0.170 ± 0.014, 0.175 ± 0.013, and 0.175 ± 0.013 g in naïve, control (# *p* < 0.05), 250 mg/kg AMK extract, 500 mg/kg AMK extract (* *p* < 0.05), AMT (* *p* < 0.05), and SSZ (* *p* < 0.05), respectively; Figure 5B). Also, AMK administration significantly reduced stool scores (0.75 ± 0.96, 2.75 ± 0.50, 1.25 ± 0.50, 2.00 ± 0.82, 0.50 ± 0.58, and 1.00 ± 0.82 g in naïve, control (## *p* < 0.01), 250 mg/kg AMK extract (* *p* < 0.05), 500 mg/kg AMK extract, AMT (*** *p* < 0.001), and SSZ (** *p* < 0.01), respectively; Figure 5C). These results suggest that AMK administration normalized the colonic changes caused by zymosan-induced colitis and suppressed diarrheal symptoms.

### 3.6. Effects of AMK Extract on Body Weight Change and Food Intake

The AMK extract, particularly 250 mg/kg of AMK extract, significantly inhibited weight loss, which was sustained on days 8 and 12, indicating the efficacy of AMK in mitigating weight loss (Figure 5D). Notably, food intake was not significantly different among the various treatment groups (Figure 5E). These results suggest that AMK does not impact food intake and is efficacious in preventing weight loss in a zymosan-induced IBS mouse model.

### 3.7. Effect of AMK Extract on Tissue Changes and TNF-α Expression Levels

**Figure 6:**
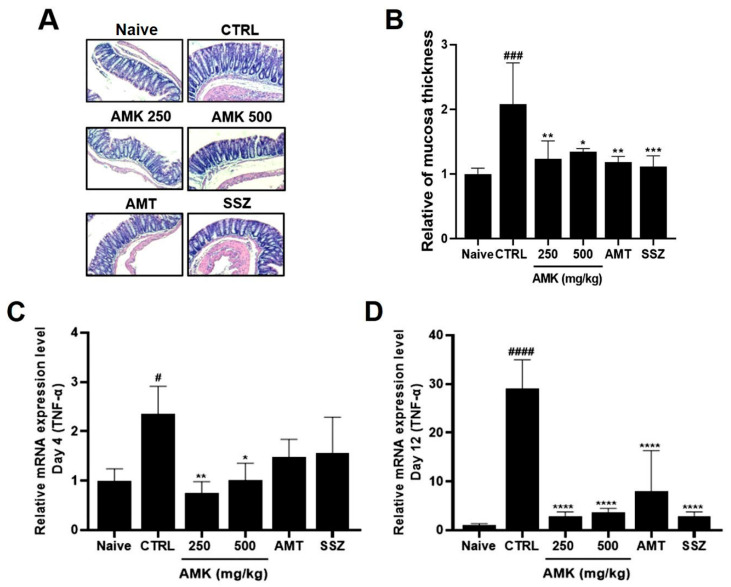
Effects of AMK extract on tissue changes and TNF-α expression levels. H&E staining reveals (**A**) histological changes and (**B**) quantitative results of colon mucosa thickness in diverse treatment groups at a 50× magnification. TNF-α expression on days (**C**) 4 and (**D**) 12 was quantified using RT-qPCR analysis. Mean ± SE. # *p* < 0.05, ### *p* < 0.001 and #### *p* < 0.0001 denote statistical significance in comparison to the naïve, while * *p* < 0.05, ** *p* < 0.01, *** *p* < 0.001, and **** *p* < 0.0001 indicate statistical significance relative to the control. CTRL: Control.

H&E staining revealed notable histological alterations in the colons of the control mice with IBS, exhibiting an increased tissue thickness compared to that in the normal mice. Inflammation was evident in the colons of the zymosan-induced mice. In particular, the tissue thickness of the AMK-extract-administered mice showed a concentration-dependent recovery to levels similar to those in the normal control group (Figure 6A,B). Colon tissues from the mice were obtained during autopsy on days 4 and 12 after colitis induction with zymosan. The expression level of TNF-α, which serves as an inflammation indicator in tissues, was determined using RT-qPCR analysis. The control group showed a noticeably higher expression of TNF-α both on days 4 and 12, indicating a significant increase in the level of TNF-α associated with inflammation. However, the analysis showed that the TNF-α levels statistically significantly decreased with 250 and 500 mg/kg AMK extract on days 4 and 12. On days 12, the expression level was reduced in both the AMK extract and positive control groups (Figure 6C,D). These results suggest that AMK had the potential inhibitory effects of GI inflammation in the zymosan-induced IBS model.

### 3.8. Effects of AMK Extract on Pain-Related Behaviors

**Figure 7:**
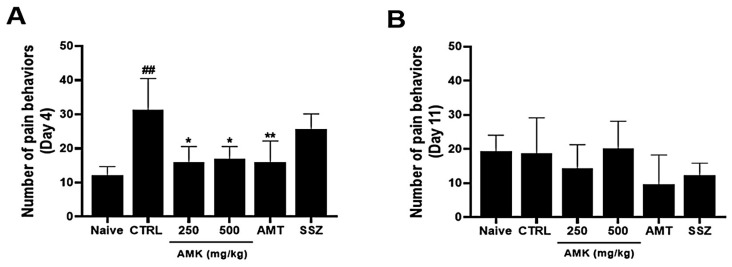
Effects of AMK extract on visceral pain-related behaviors. Pain-related behaviors were checked on days (**A**) 4 and (**B**) 11. The results are represented as mean ± SE. ## *p* < 0.01 denotes statistical significance in comparison to the naïve, while * *p* < 0.05 and ** *p* < 0.01 indicate statistical significance relative to the control. CTRL: Control.

On day 4, the average number of pain-related behaviors increased. The average numbers of behaviors with the AMK extract were decreased in visceral pain (Figure 7A). On day 11, pain-related behavior did not persist in the control group, and the average number of pain-related behaviors was not significantly different among different AMK extracts (Figure 7B).

### 3.9. Effects of AMK Extract on TRP Channel Currents

**Figure 8:**
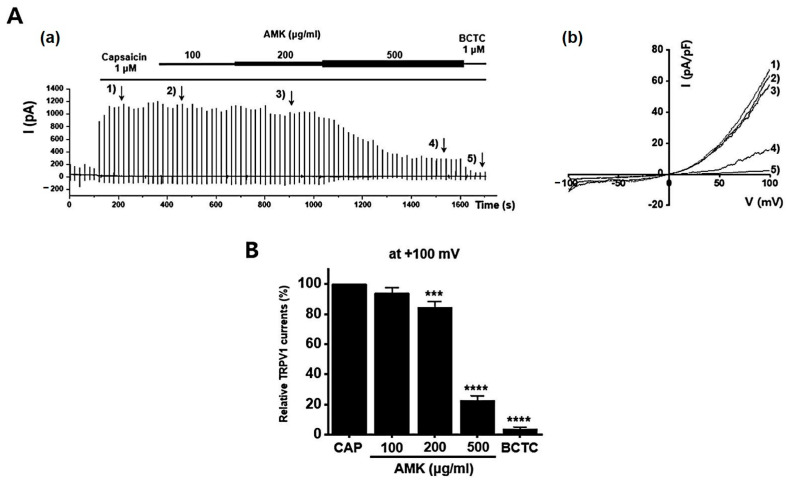
Effects of AMK extract on TRPV1 currents. (**A**) (**a**) Representative trace for TRPV1 showing the effect of 100, 200, and 500 μg/mL AMK extract. TRPV1 was overexpressed in HEK293T cells. (**b**) I–V representative curve showing the effect of 100, 200, and 500 μg/mL AMK extract. (**B**) Statistical analysis of normalized fold changes. After inward I_TRPV1_ establishment using capsaicin, relative I_TRPV1_ using 100, 200, and 500 μg/mL AMK extract and the inhibitor BCTC are shown (at +100 mV). The results are represented as mean ± SE. *** *p* < 0.001 and **** *p* < 0.0001 indicate statistical significance relative to the naïve group. AMK: *Atractylodes macrocephala* Koidz.

**Figure 9:**
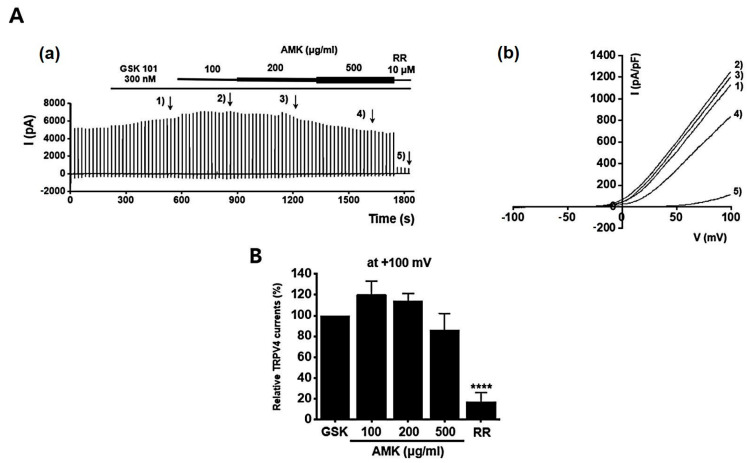
Effects of AMK extract on TRPV4 currents. (**A**) (**a**) Representative trace for TRPV4 showing the effect of 100, 200, and 500 μg/mL AMK extract. TRPV4 was overexpressed in HEK293T cells. (**b**) I–V representative curve showing the effect of 100, 200, and 500 μg/mL AMK extract. (**B**) Statistical analysis. After inward I_TRPV4_ establishment using GSK101A, relative I_TRPV4_ using 100, 200, and 500 μg/mL AMK extract and the inhibitor RR are shown (at +100 mV). The results are represented as mean ± SE. **** *p* < 0.0001 indicates statistical significance relative to the naïve.

**Figure 10:**
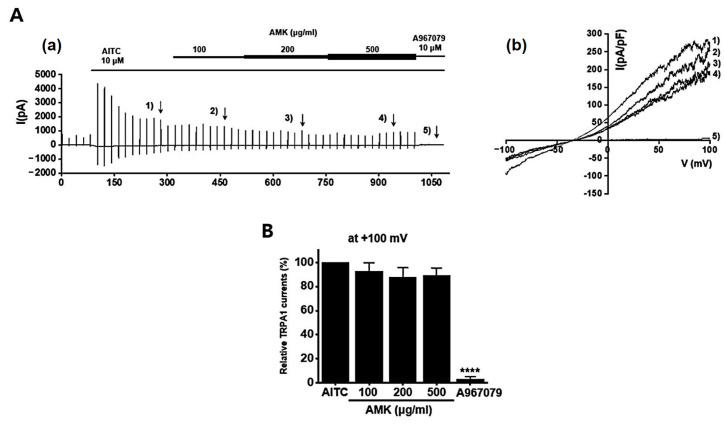
Effects of AMK extract on TRPA1 currents. (**A**) (**a**) Representative trace for TRPA1 showing the effect of 100, 200, and 500 μg/mL AMK extract. (**b**) I–V representative curve showing the effect of 100, 200, and 500 μg/mL AMK extract. (**B**) Statistical analysis. After inward I_TRPA1_ establishment using AITC, relative I_TRPA1_ using 100, 200, and 500 μg/mL AMK extract and the inhibitor A967079 are shown (at +100 mV). The results are represented as mean ± SE. **** *p* < 0.0001 indicates statistical significance relative to the naïve.

To investigate the potential regulatory effects of AMK on TRPV1 channels, we conducted whole-cell electrophysiological recordings of TRPV1-overexpressing HEK293T cells. We generated current–voltage (I–V) curves at specific time points by applying a ramp pulse from −100 mV to 100 mV under different conditions: control, 100 μg/mL AMK extract, 200 μg/mL AMK extract, and 500 μg/mL AMK extract. BCTC, a selective TRPV1 inhibitor, was used to confirm the relevance of TRPV1 [20]. The bath application of 1 μM capsaicin activated the TRPV1 current (I_TRPV1_) [21]. Interestingly, 100 μg/mL of AMK extract did not show significant changes, whereas 200 μg/mL (*** *p* < 0.001) and 500 μg/mL (**** *p* < 0.0001) of AMK extract significantly decreased I_TRPV1_ (Figure 8A). The normalized current amplitude at +100 mV is plotted for each concentration (Figure 8B). To investigate whether AMK regulated the TRPV4 channels, we conducted whole-cell electrophysiological analyses of TRPV4-overexpressing HEK293T cells. Ruthenium red (RR), a selective TRPV4 inhibitor, was used to confirm the relevance of TRPV4 [22]. The bath application of 300 nM GSK101A activated the TRPV4 current (I_TRPV4_) [23]. However, the application of 100, 200, and 500 μg/mL of AMK extract had no effects on I_TRPV4_ (Figure 9A). The normalized current amplitude at +100 mV is plotted for each concentration (Figure 9B). To investigate whether AMK regulated the TRPA1 channels, we conducted whole-cell electrophysiological analyses of TRPA1-overexpressing HEK293T cells. A967079, a selective TRPA1 inhibitor, was used to confirm the relevance of TRPA1 [24]. The bath application of 100 μM AITC activated the TRPA1 current (I_TRPA1_) [25]. However, the application of 100, 200, and 500 μg/mL of AMK extract had no effects on I_TRPA1_ (Figure 10A). The normalized current amplitude at +100 mV is plotted for each concentration (Figure 10B). These results suggest that TRPV1 may be the primary factor responsible for the suppression of IBS-induced visceral hypersensitivity.

### 3.10. Effects of AMK Extract on NaV1.5 and NaV1.7 Currents

**Figure 11:**
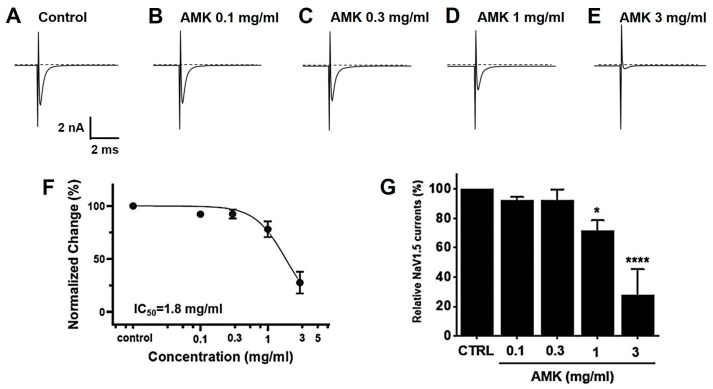
Effects of AMK extract on NaV1.5 currents. (**A**) Representative plot showing the NaV1.5 currents in HEK293T cells. (**B**) Representative plot showing the effects of 0.1 mg/mL AMK extract. (**C**) Representative plot showing the effects of 0.3 mg/mL AMK extract. (**D**) Representative plot showing the effects of 1 mg/mL AMK extract. (**E**) Representative plot showing the effects of 3 mg/mL AMK extract. (**F**) Inhibition of NaV1.5 by AMK extract. (**G**) Statistical analysis. IC_50_ = 1.8 mg/mL. The results are represented as mean ± SE. * *p* < 0.05 and **** *p* < 0.0001 indicate statistical significance relative to the control group. CTRL: Control.

**Figure 12:**
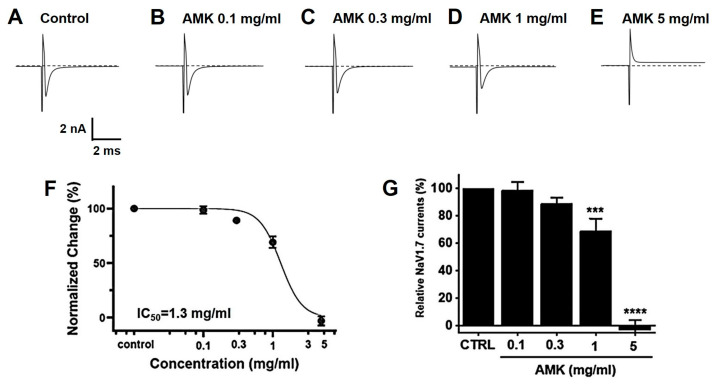
Effects of AMK extract on NaV1.7 currents. (**A**) Representative plot showing the NaV1.7 currents in HEK293T cells. (**B**) Representative plot showing the effects of 0.1 mg/mL AMK extract. (**C**) Representative plot showing the effects of 0.3 mg/mL AMK extract. (**D**) Representative plot showing the effects of 1 mg/mL AMK extract. (**E**) Representative plot showing the effects of 3 mg/mL AMK extract. (**F**) Inhibition of NaV1.7 by AMK extract. (**G**) Statistical analysis. IC_50_ = 1.3 mg/mL. The results are represented as mean ± SE. *** *p* < 0.001 and **** *p* < 0.0001 indicate statistical significance relative to the control group. CTRL: Control.

Overexpressed NaV1,5 and 1.7 currents showed rapidly decaying transient inward currents. In the NaV1.5 currents, the AMK extract reduced the peak inward current by 92.27 ± 2.18% (0.1 mg/mL), 92.47 ± 7.22% (0.3 mg/mL), 71.40 ± 7.28% (1 mg/mL), and 27.53 ± 17.79% (3 mg/mL), with an IC_50_ of 1.8 mg/mL (Figure 11). Also, in the NaV1.7 currents, the AMK extract significantly inhibited the peak inward current by 98.63 ± 6.05% (0.1 mg/mL), 89.13 ± 4.15% (0.3 mg/mL), 68.97 ± 9.29% (1 mg/mL), and −3.03 ± 7.27% (5 mg/mL), with an IC_50_ of 1.3 mg/mL (Figure 12). These results suggest that the NaV1.5 and 1.7 currents may among the factors that suppress visceral hypersensitivity due to IBS.

## 4. Discussion

IBS is a complex and multifactorial GI disorder that significantly affects the quality of life of affected individuals [26,27]. The exact pathological mechanisms underlying IBS remain poorly understood, but are believed to involve a combination of psychological, central nervous system, neuroendocrine, GI motility, and abdominal hypersensitivity factors [28]. IBS exhibits distinct subtypes based on clinical characteristics [29]. Furthermore, IBS classification can be influenced by the composition of intestinal microorganisms. The human intestine is a highly intricate ecosystem hosting a diverse array of microorganisms, including bacteria, viruses, and eukaryotes [30].

AMK, a perennial herb of the *Atractylodes* genus, has been used for over 700 years in the temperate and subtropical regions of Korea, China, and Japan [31,32]. Specifically, its rhizome has been employed for millennia to address a broad spectrum of ailments, including GI discomfort and various health issues. This versatile herb has diverse biological activities, such as enhancing GI function and exhibiting anti-tumor, anti-inflammatory, antiaging, antioxidant, antibacterial, and neuroprotective properties [33,34].

This study demonstrated that zymosan administration caused colonic changes, including a decreased colon length, increased colon weight, and worsened stool conditions, indicative of colitis. In particular, 250 and 500 mg/kg AMK extract administration normalized the colon length, reduced the colon weight, and improved the stool scores, effectively coping with colitis symptoms. Zymosan administration caused weight loss in the control rats. However, food intake was not significantly different among the different treatment groups, and 250 mg/kg AMK extract administration prevented this weight loss, indicating that it effectively preserved the body weight of the zymosan-treated rats. As observed in the histological analysis using H&E staining, tissue changes, such as an increased tissue thickness in the control mice, were restored to normal levels. An analysis of TNF-α expression, an inflammation indicator, showed that the TNF-α level was elevated in the control mice 4 and 12 days after zymosan-induced colitis. In contrast, AMK administration statistically significantly decreased TNF-α expression levels, indicating a potential anti-inflammatory effect. AMK extract reduced pain-related behaviors, especially on day 4 of administration. These results suggest that AMK may play a potential therapeutic role in alleviating IBS-associated colonic changes.

A prominent aspect of IBS research is the growing interest in the role of ion channels and their modulation in the management of IBS symptoms. TRP and NaV channels have been identified as potential targets for IBS treatment. TRPV1, TRPV4, and TRPA1 have been implicated in pain perception and immune responses within the GI tract [35]. Moreover, NaV1.5 and NaV1.7 are involved in pain perception and intestinal motility [36]. This study investigated the pharmacological effects of AMK on these channels in HEK293T cells, which play essential roles in IBS. The results demonstrated that AMK inhibited both the NaV1.5 and NaV1.7 currents in a concentration-dependent manner, indicating its potential as a therapeutic agent for IBS management. AMK significantly reduced the peak inward currents in both channels, suggesting its effectiveness in regulating GI motility and alleviating the abdominal pain associated with IBS. Among the ion channels, TRPV1, TRPV4, and TRPA1 are associated with hypersensitivity in the gut [35,37]. TRPV1 plays an important role in colorectal hypersensitivity and is, therefore a pharmacological target in IBS [38]. The dominant GI NaV channels, NaV1.5 and NaV1.7, have received substantial attention as pain inhibitors [37]. The suppression of NaV1.5 has been involved in normal GI motility restoration in patients with IBS [39], and NaV1.7 is known to be related to treatment in the case of IBS-D. Furthermore, AMK also affects the TRP channels, such as TRPV1, which are closely linked to visceral hypersensitivity. TRPV1 regulation by AMK suggests its potential role in reducing inflammation and pain perception in the colorectum, making AMK a promising candidate for IBS treatment. These findings suggest that AMK may show its therapeutic effects in IBS by modulating these ion channels, ultimately reducing pain and improving GI motility, which may contribute to its effectiveness in managing IBS. In conclusion, the results of this study highlight the potential of AMK as a promising candidate for IBS treatment. The modulation of ion channels, including NaV1.5, NaV1.7, and TRPV1, demonstrates the multifaceted approach that AMK may offer to address the diverse symptoms and pathophysiological mechanisms associated with IBS. Further research is warranted to elucidate the exact mechanisms of this and optimize AMK’s use as a traditional medicinal treatment for IBS, ultimately providing relief and improving the quality of life for individuals suffering from this challenging GI condition. Clinical trials in humans are needed to confirm the efficacy and safety of AMK in IBS treatment.

## 5. Conclusions

This study highlights the potential of AMK as a promising multifaceted therapeutic candidate for IBS management. The observed regulation of ion channels, particularly NaV1.5, NaV1.7, and TRPV1, suggests that AMK uses different approaches for addressing the symptoms and underlying mechanisms associated with IBS. These findings highlight the need for further studies to elucidate the precise molecular mechanisms by which AMK exerts its therapeutic effects. Additionally, optimizing AMK use in traditional medicinal treatments for IBS is essential for providing effective relief and improving the quality of life of individuals struggling with this complex GI disease. Although this study was conducted using a mouse model, further clinical trials in humans are needed to confirm the efficacy and safety of AMK in IBS treatment. These trials will play an important role in translating these promising findings into clinical practice and ultimately benefiting patients with IBS.

## Figures and Tables

**Table 1:** Active compounds of AMK.

Molecule Name	Structure	MW *	OB (%) *	Caco-2 *	DL *
12-senecioyl-2E,8E,10E-atractylentriol	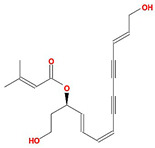	312.39	62.4	0.01	0.22
14-acetyl-12-senecioyl-2E,8E,10E-atractylentriol	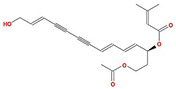	355.44	60.31	0.33	0.31
14-acetyl-12-senecioyl-2E,8Z,10E-atractylentriol	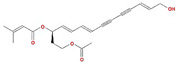	356.45	63.37	0.42	0.3
α-Longipinene	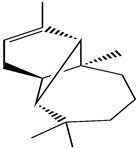	204.39	53.26	1.83	0.12
α-Amyrin	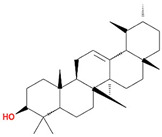	426.8	39.51	1.42	0.76
(3S,8S,9S,10R,13R,14S,17R)-10,13-dimethyl-17-[(2R,5S)-5-propan-2-yloctan-2-yl]-2,3,4,7,8,9,11,12,14,15,16,17-dodecahydro-1H-cyclopenta[a]phenanthren-3-ol	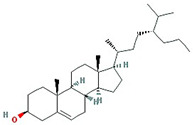	428.82	36.23	1.45	0.78
Akridin	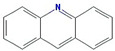	179.23	33.71	1.63	0.1
Atractylenolide I	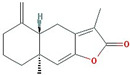	230.33	37.37	1.3	0.15
Atractylenolide II	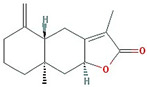	232.35	47.5	1.3	0.15
Atractylenolide III	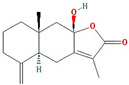	248.35	68.11	0.75	0.17
atractylone	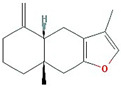	216.35	41.1	1.76	0.13
Juniper camphor	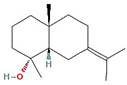	222.41	33.3	1.44	0.1
(5E,9Z)-3,6,10-trimethyl-4,7,8,11-tetrahydrocyclodeca[b]furan	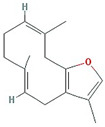	216.35	43.17	1.77	0.1
3β-acetoxyatractylone	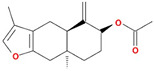	274.39	54.07	1.13	0.22
DIBP	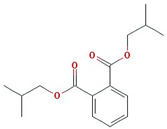	278.38	49.63	0.85	0.13
Selina-4(14),7(11)-dien-8-one	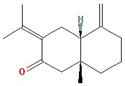	218.37	32.31	1.42	0.1
Alloaromadedrene	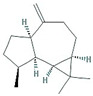	204.39	53.46	1.83	0.1
8β-ethoxy atractylenolide Ⅲ	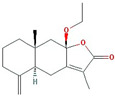	276.41	35.95	1.08	0.21

* Molecular weight (MW), oral bioavailability (OB), Caco-2 permeability (Caco-2), and drug likeness (DL).

**Table 2:** Compounds and Targets Related to GI Disease.

Molecular Name	Gene Name	Disease Name
(+/−)−Isoborneol	PTGS2	Adenomatous polyposis
		Colorectal cancer
		Peutz–Jeghers syndrome
(3S)−3−[(1R)−1,5−dimethylhex−4−enyl]−6− methylenecyclohexene	PTGS2	Adenomatous polyposis
		Colorectal cancer
		Peutz–Jeghers syndrome
(5E,9Z)−3,6,10−trimethyl−4,7,8,11−tetrahydrocyclodeca[b]furan	NOS3	Colon cancer
	PTGS2	Adenomatous polyposis
		Colorectal cancer
		Peutz–Jeghers syndrome
	SLC6A4	* Irritable bowel syndrome
14−acetyl−12−senecioyl−2E,8Z,10E−atractylentriol	PTGS2	Adenomatous polyposis
		Colorectal cancer
		Peutz–Jeghers syndrome
2−[(1R,3S,4S)−3−isopropenyl−4−methyl−4−vinylcyclohexyl]propan−2−ol	SLC6A4	* Irritable bowel syndrome
3β−acetoxyatractylone	OPRM1	Diarrhea
		Opioid-induced bowel dysfunction
		* Irritable bowel syndrome
	NOS3	Colon cancer
		Adenomatous polyposis
	PTGS2	Colorectal cancer
		Peutz–Jeghers syndrome
	SCN5A	* Irritable bowel syndrome
8β−ethoxy atractylenolide Ⅲ	PTGS2	Adenomatous polyposis
		Colorectal cancer
		Peutz–Jeghers syndrome
Akridin	PTGS2	Adenomatous polyposis
		Colorectal cancer
		Peutz–Jeghers syndrome
Alpha−Curcumene	PTGS2	Adenomatous polyposis
		Colorectal cancer
		Peutz–Jeghers syndrome
Alpha−humulene	PTGS2	Adenomatous polyposis
		Colorectal cancer
		Peutz–Jeghers syndrome
	TNF	* Irritable bowel syndrome
ASI	ALOX5	Gastrointestinal Cancers
		Inflammatory Bowel Disease
		Pancreatic Cancer
		Ulcerative colitis
Atractylenolide i	TNF	* Irritable bowel syndrome
Atractylenolide iii	TNF	* Irritable bowel syndrome
Atractylone		Diarrhea
	OPRM1	Opioid-induced bowel dysfunction
		* Irritable bowel syndrome
	NOS3	Colon cancer
	SCN5A	* Irritable bowel syndrome
Beta−caryophyllene	PTGS2	Adenomatous polyposis
		Colorectal cancer
		Peutz–Jeghers syndrome
Beta−Humulene	PTGS2	Adenomatous polyposis
		Colorectal cancer
		Peutz–Jeghers syndrome
Beta−Selinene	PTGS2	Adenomatous polyposis
		Colorectal cancer
		Peutz–Jeghers syndrome
D−Camphene		Adenomatous polyposis
	PTGS2	Colorectal cancer
		Peutz–Jeghers syndrome
	SCN5A	* Irritable bowel syndrome
DIBP	SLC6A4	* Irritable bowel syndrome
DTY	NOS3	Colon cancer
		Adenomatous polyposis
	PTGS2	Colorectal cancer
		Peutz–Jeghers syndrome
GLY	CTNNB1	Colorectal cancer
	LTA4H	Esophageal cancer
		Crohns’s Disease, unspecified
	MMP12	Gastro-intestinal ulcers
		Ulcerative colitis
	AMY2A	Pancreatic disease
		Adenomatous polyposis
	PTGS2	Colorectal cancer
		Peutz–Jeghers syndrome
	RRM1	Pancreatic Neoplasms
Gulutamine	LTA4H	Esophageal cancer
		Adenomatous polyposis
	PTGS2	Colorectal cancer
		Peutz–Jeghers syndrome
Hemo−sol	PTGS2	Adenomatous polyposis
		Colorectal cancer
		Peutz–Jeghers syndrome
Istidina	PTGS2	Adenomatous polyposis
		Colorectal cancer
		Peutz–Jeghers syndrome
Juniper camphor	PTGS2	Adenomatous polyposis
		Colorectal cancer
		Peutz–Jeghers syndrome
L−Arginin	PTGS2	Adenomatous polyposis
		Colorectal cancer
		Peutz–Jeghers syndrome
LPG		Crohns’s Disease, unspecified
	MMP12	Gastro-intestinal ulcers
		Ulcerative colitis
	RRM1	Pancreatic Neoplasms
L−Valin	PTGS2	Adenomatous polyposis
		Colorectal cancer
		Peutz–Jeghers syndrome
Palmitic acid	PTGS2	Adenomatous polyposis
		Colorectal cancer
		Peutz–Jeghers syndrome
	TNF	* Irritable bowel syndrome
PHA	NOS3	Colon cancer
		Adenomatous polyposis
	PTGS2	Colorectal cancer
		Peutz–Jeghers syndrome
	SCN5A	* Irritable bowel syndrome
Prolinum	PTGS2	Adenomatous polyposis
		Colorectal cancer
		Peutz–Jeghers syndrome
Scopoletol	CA1	Pancreatic cancer
	LTA4H	Esophageal cancer
		Adenomatous polyposis
	PTGS2	Colorectal cancer
		Peutz–Jeghers syndrome
Selina−4(14),7(11)−dien−8−one	PTGS2	Adenomatous polyposis
		Colorectal cancer
		Peutz–Jeghers syndrome
α−Longipinene	PTGS2	Adenomatous polyposis
		Colorectal cancer
		Peutz–Jeghers syndrome
γ−elemene	PTGS2	Adenomatous polyposis
		Colorectal cancer
		Peutz–Jeghers syndrome

* After investigating the relationship between AMK and IBS using cytoscape stringApp, genes related to IBS were added to this table.

## Data Availability

The original data are available upon reasonable request to the corresponding author. The data are not publicly available due to privacy and ethical restrictions.

## Supplementary Materials

The following supporting information can be downloaded at: https://www.mdpi.com/article/10.3390/nu16111683/s1, Table S1: 100 IBS-related genes.
