# Supplementary material for: Atractylodes macrocephala Koidz Alleviates Symptoms in Zymosan-Induced Irritable Bowel Syndrome Mouse Model through TRPV1, NaV1.5, and NaV1.7 Channel Modulation
Source: Nutrients. 2024 May 29;16(11):1683. doi: 10.3390/nu16111683 (PMC11174792; doi:10.3390/nu16111683)
Supplement: Supplementary file 1 [file nutrients-16-01683-s001.zip › nutrients-2980009-supplementary.pdf]

Supplementary Table S1.

100 IBS–related genes

Gene name	Protein name
ACTB	Actin, cytoplasmic 1
ALB	Serum albumin
AOC1	Amiloride-sensitive amine oxidase [copper-containing]
ATP12A	ATPase, H+/K+ transporting, nongastric, alpha polypeptide
ATP4A	ATPase, H+/K+ exchanging, alpha polypeptide
BDNF	Brain-derived neurotrophic factor
CACNA1H	Calcium channel, voltage-dependent, T type, alpha 1H subunit
CALCA	Calcitonin-related polypeptide alpha
CCK	Cholecystokinin
CD4	T-cell surface antigen T4/Leu-3
CFTR	Cystic fibrosis transmembrane conductance regulator (ATP-binding cassette sub-family C, member 7)
CGN	Cingulin
CHGA	Chromogranin A (parathyroid secretory protein 1)
CLCN2	Chloride channel, voltage-sensitive 2
CLDN1	Senescence-associated epithelial membrane protein
CLDN2	Claudin 2
CLDN4	Williams-Beuren syndrome chromosomal region 8 protein
CRH	Corticotropin releasing hormone
CRHR1	Corticotropin releasing hormone receptor 1
CRHR2	Corticotropin releasing hormone receptor 2
CRP	C-reactive protein, pentraxin-related
CXCL8	Monocyte-derived neutrophil chemotactic factor
CYP7A1	Cytochrome P450, family 7, subfamily A, polypeptide 1
F2RL1	Coagulation factor II (thrombin) receptor-like 1
FABP2	Intestinal-type fatty acid-binding protein
FFAR2	G-protein coupled receptor 43
FFAR3	G-protein coupled receptor 41
FGF19	Fibroblast growth factor 19
FGFR4	Fibroblast growth factor receptor 4
FOS	FBJ murine osteosarcoma viral oncogene homolog
GAPDH	Glyceraldehyde-3-phosphate dehydrogenase
GAST	Gastrin
GCG	Glucagon
GHRL	Growth hormone-releasing peptide

GOT2	Plasma membrane-associated fatty acid-binding protein
GPBAR1	G protein-coupled bile acid receptor 1
GPSM2	G-protein signaling modulator 2
GRID2IP	Glutamate receptor, ionotropic, delta 2 (Grid2) interacting protein
GUCA2A	Guanylate cyclase activator 2A (guanylin)
GUCA2B	Guanylate cyclase activator 2B (uroguanylin)
GUCY2C	Guanylate cyclase 2C (heat stable enterotoxin receptor)
HCAR2	Hydroxycarboxylic acid receptor 2
HP	Haptoglobin
HRH1	Histamine H1 receptor
HTR1A	5-hydroxytryptamine (serotonin) receptor 1A, G protein-coupled
HTR2B	5-hydroxytryptamine (serotonin) receptor 2B, G protein-coupled
HTR3A	5-hydroxytryptamine (serotonin) receptor 3A, ionotropic
HTR3B	5-hydroxytryptamine (serotonin) receptor 3B, ionotropic
HTR3C	5-hydroxytryptamine (serotonin) receptor 3C, ionotropic
HTR3E	5-hydroxytryptamine (serotonin) receptor 3E, ionotropic
HTR4	5-hydroxytryptamine (serotonin) receptor 4, G protein-coupled
IL10	Cytokine synthesis inhibitory factor
IL13	Interleukin 13
IL17A	Cytotoxic T-lymphocyte-associated antigen 8
IL18	Interferon gamma-inducing factor
IL1B	Interleukin 1, beta
IL4	Lymphocyte stimulatory factor 1
IL6	B-cell stimulatory factor 2
KDELR2	KDEL (Lys-Asp-Glu-Leu) endoplasmic reticulum protein retention receptor 2
KLB	Klotho beta-like protein
LCT	Lactase-phlorizin hydrolase
LRP5	Low density lipoprotein receptor-related protein 5
MLN	Promotilin
MPO	Myeloperoxidase
MYLK	Myosin light chain kinase, smooth muscle
NGF	Nerve growth factor (beta polypeptide)
NLRP6	NACHT, LRR and PYD domains-containing protein 6
NPR3	Atrial natriuretic peptide clearance receptor
NPY	Pro-neuropeptide Y
NR1H4	Nuclear receptor subfamily 1, group H, member 4
NR1I2	Nuclear receptor subfamily 1, group I, member 2
NXPH1	Neurexophilin 1
OCLN	Occludin

OPRM1	Mu-type opioid receptor
P2RX3	Purinergic receptor P2X, ligand-gated ion channel, 3
PDZD3	Natrium-phosphate cotransporter IIa C-terminal-associated protein 2
POMC	Corticotropin-lipotropin
PYY	Peptide tyrosine tyrosine
SCN5A	Sodium channel, voltage-gated, type V, alpha subunit
SLC10A2	Solute carrier family 10 (sodium/bile acid cotransporter), member 2
SLC6A4	Solute carrier family 6 (neurotransmitter transporter), member 4
SLC9A3	Solute carrier family 9, subfamily A (NHE3, cation proton antiporter 3), member 3
SST	Growth hormone release-inhibiting factor
TAC1	Tachykinin, precursor 1
TACR2	Neurokinin A receptor
TGM2	Protein-glutamine gamma-glutamyltransferase 2
TJP1	Tight junction protein ZO-1
TJP3	Tight junction protein ZO-3
TLR2	Toll/interleukin-1 receptor-like protein 4
TLR4	Toll-like receptor 4
TLR5	Toll/interleukin-1 receptor-like protein 3
TNF	Tumor necrosis factor ligand superfamily member 2
TNFSF15	Tumor necrosis factor (ligand) superfamily, member 15
TOR1A	Torsin family 1, member A (torsin A)
TPH1	Tryptophan hydroxylase 1
TRPA1	Transient receptor potential cation channel, subfamily A, member 1
TRPM8	Transient receptor potential cation channel, subfamily M, member 8
TRPV4	Transient receptor potential cation channel, subfamily V, member 4
VIP	Vasoactive intestinal peptide
VR1	Transient receptor potential cation channel, subfamily V, member 1
